# Drowsy Driving and Risk Behaviors — 10 States and Puerto Rico, 2011–2012

**Published:** 2014-07-04

**Authors:** Anne G. Wheaton, Ruth A. Shults, Daniel P. Chapman, Earl S. Ford, Janet B. Croft

**Affiliations:** 1 Division of Population Health, National Center for Chronic Disease Prevention and Health Promotion; 2 Division of Unintentional Injury Prevention, National Center for Injury Prevention and Control, CDC

Findings in published reports have suggested that drowsy driving is a factor each year in as many as 7,500 fatal motor vehicle crashes (approximately 25%) in the United States (1,2). CDC previously reported that, in 2009–2010, 4.2% of adult respondents in 19 states and the District of Columbia reported having fallen asleep while driving at least once during the previous 30 days (3). Adults who reported usually sleeping ≤6 hours per day, snoring, or unintentionally falling asleep during the day were more likely to report falling asleep while driving compared with adults who did not report these sleep patterns (3). However, limited information has been published on the association between drowsy driving and other risk behaviors that might contribute to crash injuries or fatalities. Therefore, CDC analyzed responses to survey questions regarding drowsy driving among 92,102 respondents in 10 states and Puerto Rico to the 2011–2012 Behavioral Risk Factor Surveillance System (BRFSS) surveys. The results showed that 4.0% reported falling asleep while driving during the previous 30 days. In addition to known risk factors, drowsy driving was more prevalent among binge drinkers than non-binge drinkers or abstainers and also more prevalent among drivers who sometimes, seldom, or never wear seatbelts while driving or riding in a car, compared with those who always or almost always wear seatbelts. Drowsy driving did not vary significantly by self-reported smoking status. Interventions designed to reduce binge drinking and alcohol-impaired driving, to increase enforcement of seatbelt use, and to encourage adequate sleep and seeking treatment for sleep disorders might contribute to reductions in drowsy driving crashes and related injuries.

Each year, state health departments administer BRFSS, a random-digit–dialed telephone survey of noninstitutionalized adults aged ≥18 years, in collaboration with CDC. The response rate is the number of respondents who completed the survey as a proportion of all eligible and likely eligible persons.* The median survey response rate for all 50 states and the District of Columbia, was 49.7% in 2011 (range = 33.8%–64.1%) and 45.2% in 2012 (range = 27.7%–60.4%). Questions regarding insufficient sleep were asked in an optional sleep module used by only 10 states and Puerto Rico†; therefore, this analysis was confined to those 10 states and Puerto Rico. Response rates for the 10 states and Puerto Rico had a median of 51.7% in 2011 and ranged from 35.4% (California) to 61.7% (Puerto Rico); response rates in 2012 had a median of 47.0% and ranged from 39.4% (Oregon) to 58.2% (Puerto Rico).§

A total of 92,102 respondents were asked, “During the past 30 days, have you ever nodded off or fallen asleep, even just for a brief moment, while driving?” Drowsy driving was defined as an affirmative response, whereas no drowsy driving included responses of “no” and also 81 responses of “don’t know/not sure.” Those who responded that they did not drive or did not have a license (5,575) were excluded from the analysis. Frequent insufficient sleep was defined as ≥14 days in response to “During the past 30 days, for about how many days have you felt you did not get enough rest or sleep?” Respondents were also asked: “On average, how many hours of sleep do you get in a 24-hour period? Think about the time you actually spend sleeping or napping, not just the amount of sleep you think you should get.” “Do you snore?” and “During the past 30 days, for about how many days did you find yourself unintentionally falling asleep during the day (categorized as none or ≥1 day)?”

Smoking status included current smoker, former smoker, and never smoker. Alcohol use status included binge drinker, non-binge drinker, and abstainer. Binge drinking was defined for men as having five or more drinks and for women as having four or more drinks on at least one occasion during the preceding month. Abstainers were respondents who had not consumed any alcoholic beverages during the preceding month. Respondents also were asked about their frequency of seatbelt use and categorized as “always or almost always” and “sometimes, seldom, or never” users.

The age-adjusted prevalences of falling asleep while driving (with 95% confidence intervals) were calculated by state, selected demographic characteristics, sleep-related characteristics, and risk behaviors using statistical software that took into account the complex sampling design. For comparisons of prevalence between subgroups, statistical significance (p<0.05) was determined by using t-tests. All indicated differences between subgroups are statistically significant.

Among the 92,102 respondents, 4.0% reported falling asleep while driving during the preceding 30 days (Table 1). Drowsy driving decreased with age (linear trend p<0.001) from 5.9% among adults aged 18–24 years to 1.8% among adults aged ≥65 years. Overall, the age-adjusted prevalence of drowsy driving was higher among men than women (5.0% compared with 3.0%, p<0.001). The prevalence of drowsy driving for men aged 18–34 years was 6.9%, compared with 3.5% for women in the same age group. Drowsy driving prevalence was higher among all other racial/ethnic groups compared with non-Hispanic whites (p<0.05) and did not differ by educational level. Among the 10 states and Puerto Rico, drowsy driving prevalence ranged from 1.8% in Oregon to 7.4% in Puerto Rico (Table 1). These prevalence estimates can be extrapolated to approximately 1.8 million drivers driving drowsy in the last 30 days in the 10 states and Puerto Rico included in this report.¶

Respondents who usually slept ≤5 hours per 24 hours reported drowsy driving more often than those who slept 6 hours or ≥7 hours (9.1% compared with 5.2% [p<0.001] and 2.7% [p<0.001], respectively), as did snorers compared with non-snorers (5.6% compared with 2.9%, p<0.001) (Table 2). In addition, drowsy driving was more common among binge drinkers than non-binge drinkers and abstainers (5.2% compared with 3.7% [p=0.028] and 3.6% [p=0.005], respectively). Drowsy driving also was more common among drivers who sometimes, seldom, or never wear seatbelts while driving or riding in a car compared with those who always or almost always wear seatbelts (6.6% compared with 3.9%, p=0.005). Drowsy driving did not vary by smoking status.

## Discussion

CDC has named motor vehicle injury prevention as one of its 10 “winnable battles.”** More than 30,000 persons have died in motor vehicle crashes each year since 1963 (4). In 2012, nearly one third (10,322) of the 33,561 traffic fatalities occurred in alcohol-impaired driving crashes (i.e., a driver involved in the crash had a blood alcohol content of ≥0.08 g/dL), and 70% of the alcohol-impaired drivers involved in these fatal crashes had a blood alcohol content of ≥0.15 g/dL, indicating binge drinking.†† In addition, half of vehicle occupants killed were not wearing seatbelts (4), and as many as 7,500 fatal crashes in the United States each year might involve drowsy drivers (1,2).

Effective interventions exist to address binge drinking, alcohol-impaired driving, and nonuse of seatbelts. Information about these interventions has been published by the Community Preventive Services Task Force.§§ This study showed that drivers who reported binge drinking or infrequent (sometimes, seldom, or never) use of seatbelts also were more likely to drive drowsy; therefore, enforcement efforts aimed at these behaviors might also help reduce drowsy driving crashes and resulting injuries, as well as provide opportunities for increasing awareness of the dangers of drowsy driving. Because young men are more likely to engage in all of these risk behaviors, interventions might be aimed at this high-risk population.

Falling asleep while driving is clearly dangerous, but drowsiness also impairs the ability to drive safely even if drivers do not fall asleep. Studies have observed that drowsy drivers take longer to react, are less attentive to their environment, and have impaired decision-making skills (5), all of which can contribute to vehicle crashes. Sleep-related crashes are more likely to happen at times when drivers are more likely to be sleepy: at night or in the midafternoon (6,7). Although these crashes often involve a single vehicle going off the road, sleep-related crashes also are disproportionately represented in rear-end and head-on collisions. Finally, injuries and fatalities are more common in drowsy driving crashes than non-drowsy driving crashes (6). Various technologies have been developed to prevent drowsy driving crashes (7). Detection technologies use in-vehicle devices to sense changes in the driver that indicate sleepiness, such as excessive eyelid closure and head-nodding. Other technologies, such as the use of rumble strips (shoulder or center line), in-vehicle lane departure warning systems, and collision avoidance systems, are designed to prevent crashes from driver fatigue or inattention. Although evaluation of the effectiveness of in-vehicle technologies in preventing crashes is in the early stages, results to date are promising (7,8).

What is already known on this topic?

As many as 7,500 fatal motor vehicle crashes in the United States each year might involve drowsy driving, and 4.2% of adult respondents to a 2009–2010 survey reported falling asleep while driving at least once during the previous 30 days. Adults who reported usually sleeping ≤6 hours per day, snoring, or unintentionally falling asleep during the day were more likely to report falling asleep while driving than adults who did not.

What is added by this report?

CDC analyzed data regarding drowsy driving by selected characteristics, including sleep patterns and risk behaviors, from 92,102 adult survey respondents in 10 states and Puerto Rico in 2011–2012. Among the respondents, 4% reported having fallen asleep while driving in the previous 30 days. In addition to known risk factors, drowsy driving was more prevalent among men, younger drivers, binge drinkers, and among drivers who did not regularly use seatbelts compared with other respondents.

What are the implications for public health practice?

Interventions designed to reduce binge drinking and alcohol-impaired driving, to enforce seatbelt use, and to encourage adequate sleep and seeking treatment for sleep disorders might contribute to reductions in drowsy driving crashes and their related deaths and injuries.

The findings in this report are subject to at least three limitations. First, estimates of falling asleep while driving are based on self-report, which likely result in underestimates. Persons often are not aware that they have fallen asleep, even after several minutes asleep (9). Second, data were not collected for all states and might not be generalizable to the rest of the United States. In addition, because response rates for the states that used the optional sleep module during 2011–2012 were relatively low, ranging from 35.4% to 60.9% (median = 51.7%), nonresponse bias might have affected the results. Finally, BRFSS does not survey persons aged <18 years, thereby excluding young drivers who might be at increased risk for drowsy driving (6).

To prevent drowsy driving, drivers should get enough sleep (7–8 hours for adults), seek treatment for sleep disorders, and avoid alcohol use before driving. Even small amounts of alcohol can amplify driver impairment caused by drowsiness (10). Drivers should recognize the symptoms of drowsiness and respond appropriately when on the road. Symptoms of drowsiness include frequent yawning or blinking, difficulty remembering the past few miles driven, missing exits, drifting from a lane, or hitting a rumble strip. Drivers are advised to get off the road and rest until no longer drowsy or change drivers if they experience these symptoms. Turning up the radio, opening the window, and turning up the air conditioner have not proven to be effective techniques to stay awake (7). Public health professionals in motor vehicle injury prevention can learn about drowsy driving countermeasures and other highway safety countermeasures in the National Highway Traffic Safety Administration’s guide *Countermeasures That Work*.¶¶

## Figures and Tables

**TABLE 1:** Age-adjusted* prevalence of falling asleep while driving in the preceding 30 days among drivers aged ≥18 years, by selected characteristics — Behavioral Risk Factor Surveillance System, 10 states and Puerto Rico, 2011–2012

Characteristic	No.†	No. who reported falling asleep while driving	%§	(95% CI)
**Total**	**92,102**	**2,602**	**4.0**	**(3.7–4.4)**
**Sex**				
Men	37,105	1,368	5.0	(4.4–5.7)
Women	54,997	1,234	3.0	(2.6–3.5)
**Age group (yrs)**				
18–24	3,885	179	5.9	(4.4–8.0)
25–34	8,365	353	4.8	(3.8–6.0)
35–44	12,177	507	4.4	(3.7–5.3)
45–54	17,359	592	4.2	(3.5–5.0)
55–64	21,519	564	2.9	(2.3–3.5)
≥65	28,797	407	1.8	(1.5–2.3)
**Race/Ethnicity**				
White, non-Hispanic	70,783	1,605	2.9	(2.6–3.3)
Black, non-Hispanic	2,595	110	7.0	(4.9–9.9)
Hispanic	12,678	704	4.9	(4.2–5.7)
Other race¶, non-Hispanic	5,425	167	6.5	(4.8–8.7)
**Education level**				
Less than high school diploma or GED	6,701	199	3.6	(2.6–5.1)
High school diploma or GED	24,633	655	4.1	(3.4–5.0)
At least some college	60,628	1,743	4.1	(3.7–4.7)
**Employment status**				
Employed	46,866	1,725	4.5	(4.0–5.1)
Unemployed	5,320	170	3.7	(2.6–5.1)
Retired	25,997	339	**	
Unable to work	5,080	182	**	
Student/Homemaker	8,624	185	2.5	(1.9–3.3)
**Location of residence**				
Alaska	7,025	124	2.1	(1.6–2.7)
California	9,314	303	4.5	(3.8–5.2)
Kansas	13,306	349	3.6	(3.1–4.1)
Maine	3,658	75	3.7	(2.6–5.3)
Massachusetts	5,531	141	3.3	(2.4–4.5)
Minnesota	13,535	353	3.1	(2.6–3.6)
Nebraska	9,461	282	3.2	(2.6–3.9)
Nevada	8,039	182	2.8	(2.3–3.4)
Oregon	9,253	150	1.8	(1.4–2.3)
Tennessee	4,917	126	3.8	(2.6–5.5)
Puerto Rico	8,063	517	7.4	(6.6–8.2)
**Median (range)**			**3.3**	**(1.8–7.4)**

**Abbreviations:** CI = confidence interval; GED = General Educational Development certificate.

* Age adjusted to the 2000 projected U.S. population, except for age groups.

† Unweighted sample. Categories might not sum to survey total because of missing responses.

§ Weighted percentage.

¶ Asian, Native Hawaiian or Pacific Islander, American Indian/Alaska Native, and multiracial.

** Estimate is unreliable. Relative standard error >0.3.

**TABLE 2:** Age-adjusted* prevalence of falling asleep while driving in the preceding 30 days among drivers aged ≥18 years, by sleep patterns and risk behaviors — Behavioral Risk Factor Surveillance System, 10 states and Puerto Rico, 2011–2012†

Characteristic	No.§	No. who reported falling asleep while driving	%	(95% CI)
**Sleep patterns**				
Frequent insufficient sleep (≥14 days of insufficient rest or sleep in the preceding 30 days)				
Yes	22,711	1,139	6.2	(5.4–7.1)
No	69,279	1,462	3.2	(2.8–3.6)
Usual sleep duration (per 24 hrs)				
≤5 hrs	8,693	568	9.1	(7.5–11.2)
6 hrs	19,610	789	5.2	(4.4–6.1)
7 hrs	27,762	623	3.0	(2.4–3.7)
8 hrs	25,710	417	2.4	(1.9–3.0)
≥9 hrs	9,482	179	2.7	(1.8–3.8)
Snoring				
Yes	43,902	1,541	5.6	(4.8–6.5)
No	48,178	1,061	2.9	(2.6–3.4)
Unintentionally fell asleep during the day (≥1 day in the preceding 30 days)				
Yes	29,394	1,815	8.9	(8.0–9.9)
No	62,652	786	1.6	(1.3–1.9)
**Risk behaviors**				
Smoking status				
Current smoker	13,435	382	4.3	(3.3–5.5)
Former smoker	27,291	687	3.7	(3.0–4.6)
Never smoker	50,995	1,522	4.0	(3.6–4.5)
Alcohol use (previous 30 days)				
None (abstainers)	42,575	1,138	3.6	(3.1–4.1)
Binge drinkers¶	11,720	500	5.2	(4.3–6.3)
Non-binge drinkers**	36,588	916	3.8	(3.2–4.6)
Seatbelt use				
Always/almost always	87,175	2,361	3.9	(3.5–4.3)
Sometimes, seldom, or never	4,835	238	6.6	(5.0–8.8)

**Abbreviation:** CI = confidence interval.

* Age adjusted to the 2000 projected U.S. population.

† The sleep module was used by Alaska, California, Kansas, Maine, Massachusetts, Minnesota, Nebraska, Nevada, Oregon, Tennessee, and Puerto Rico in 2011 and again by Alaska, Kansas, Nevada, Oregon, and Puerto Rico in 2012.

§ Unweighted sample. Categories might not sum to survey total because of missing responses.

¶ Binge drinking was defined for men as having five or more drinks and for women as having four or more drinks on one occasion during the previous 30 days.

** Includes respondents who reported consuming alcohol in previous 30 days, but not binge drinking.

## Footnotes

* Additional information available at http://www.aapor.org/standard_definitions2.htm.

† Alaska, California, Kansas, Maine, Massachusetts, Minnesota, Nebraska, Nevada, Oregon, Tennessee, and Puerto Rico used the insufficient sleep module in 2011. Alaska, Kansas, Nevada, Oregon, and Puerto Rico used the module again in 2012. Aggregating data for two surveys increased the sample size in those states; sampling weights in each year were halved before obtaining the prevalence estimates in those states. The prevalence of drowsy driving was not statistically different when comparing 2011 and 2012 for the states that used the module for both years.

§ Additional information available at http://www.cdc.gov/brfss/pdf/2011_summary_data_quality_report.pdf and http://www.cdc.gov/brfss/annual_data/2012/pdf/summarydataqualityreport2012_20130712.pdf.

¶ Alaska, approximately 10,000; California, 1,052,000; Kansas, 70,000; Maine, 32,000; Massachusetts, 135,000; Minnesota, 106,000; Nebraska, 39,000; Nevada, 48,000; Oregon, 46,000; Tennessee, 130,000; Puerto Rico, 173,000.

** Additional information available at http://www.cdc.gov/winnablebattles/motorvehicleinjury.

†† Available at http://www-nrd.nhtsa.dot.gov/pubs/811870.pdf.

§§ Available at http://www.thecommunityguide.org/index.html.

¶¶ Available at http://www.nhtsa.gov/staticfiles/nti/pdf/811727.pdf.
